# Unraveling the Pivotal Roles of Various Metal Ion Centers in the Catalysis of Quercetin 2,4-Dioxygenases

**DOI:** 10.3390/molecules28176238

**Published:** 2023-08-25

**Authors:** Xueyuan Yan, Han Xiao, Jinshuai Song, Chunsen Li

**Affiliations:** 1 College of Chemistry & Chemical and Environmental Engineering, Weifang University, Weifang 261061, China; 2 State Key Laboratory of Structure of Chemistry, Fujian Institute of Research on the Structure of Matter, Chinese Academy of Sciences, Fuzhou 350002, China; 3 Institute of Green Catalysis, College of Chemistry, Zhengzhou University, Zhengzhou 450001, China; jssong@zzu.edu.cn; 4 Fujian Provincial Key Laboratory of Theoretical and Computational Chemistry, Xiamen University, Xiamen 361005, China

**Keywords:** quercetin 2,4-dioxygenase, transition metal ion co-factors, metalloenzyme, dioxygen activation, quercetin oxidative degradation, QM/MM calculation

## Abstract

Quercetin 2,4-dioxygenase (QueD) with various transition metal ion co-factors shows great differences, but the internal reasons have not been illustrated in detail. In order to explore the effects of metal ion centers on the catalytic reactivity of QueD, we calculated and compared the minimum energy crossing point (MECP) of dioxygen from the relatively stable triplet state to the active singlet state under different conditions by using the DFT method. It was found that the metal ions play a more important role in the activation of dioxygen compared with the substrate and the protein environment. Simultaneously, the catalytic reactions of the bacterial QueDs containing six different transition metal ions were studied by the QM/MM approach, and we finally obtained the reactivity sequence of metal ions, Ni^2+^ > Co^2+^ > Zn^2+^ > Mn^2+^ > Fe^2+^ > Cu^2+^, which is basically consistent with the previous experimental results. Our calculation results indicate that metal ions act as Lewis acids in the reaction to stabilize the substrate anion and the subsequent superoxo and peroxo species in the reaction, and promote the proton coupled electron transfer (PCET) process. Furthermore, the coordination tendencies of transition metal ion centers also have important effects on the catalytic cycle. These findings have general implications on metalloenzymes, which can expand our understanding on how various metal ions play their key role in modulating catalytic reactivity.

## 1. Introduction

**Figure 1:**
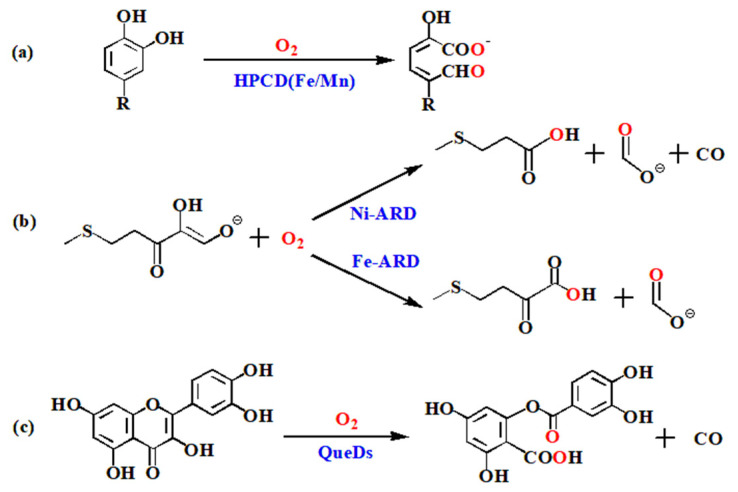
Catalytic mechanisms of three metal-containing dioxygenases: (**a**) homoprotocatechuate 2,3-dioxygenase; (**b**) acireductone dioxygenase; (**c**) quercetin 2,4-dioxygenase.

Metalloenzymes, which are widely found in animals, plants and microorganisms, can catalyze a large class of organic reactions under mild conditions and provide necessary substances for the metabolism of biological systems, playing an important role in chemical synthesis and biological transformation [1,2,3]. Metalloenzymes select metal ion cofactors as reactive centers to overcome the spin barrier to activate biological oxidants and facilitate catalytic reactions [4,5]. For example, homoprotocatechuate 2,3-dioxygenase (HPCD) with Fe^2+^ or Mn^2+^ as the reactive center can activate O_2_ to catalyze the ring opening of catecholates (Figure 1a) [6,7,8]; acireductone dioxygenase (ARD) catalyzes 1,2-dihydroxy-3-keto-5-(methylthio) pentene to obtain different products due to the different metal ions (Ni^2+^ or Fe^2+^) in the active center of the reaction (Figure 1b) [9,10,11,12]. Similarly, as a typical metalloenzyme, quercetin 2,4-dioxygenase (QueD) derived from *Streptomyces* sp. strain FLA was found to use a variety of transition metals (such as manganese, iron, cobalt, nickel, etc.) as the central metal ion cofactors to catalyze the cleavage of quercetin and other aromatic rings, and shows different catalytic activities (Figure 1c) [13,14,15]. The intrinsic effects of metal ion cofactors on enzyme catalysis were an unsolved problem in the field of metalloenzyme. Here, we take metal-containing quercetinases as the research object, and use DFT and QM/MM methods to deeply explore the intrinsic effects of various metal ions on the enzyme-catalyzed reaction.

QueD belongs to the *Cupin* protein superfamily and has a β-barrel-shaped folding structure. It uses the bivalent transition metal ion as the reactive center to form the reactive site by coordinating with three histidines and one glutamate [16,17,18]. In recent decades, a lot of experimental and theoretical studies were carried out on m-etal-containing quercetinases. Experimentally, Fetzner et al. found that the order of catalytic activity of QueDs extracted from *Streptomyces* sp. strain FLA for various metal ions was as follows: Ni^2+^ > Co^2+^ > Fe^2+^ > Mn^2+^ [19,20]. Interestingly, nickel ion, which is not often used as a central metal cofactor, showed the highest catalytic activity in QueDs, whereas iron ion, which is commonly used as a central metal cofactor in other metalloenzymes, showed relatively poor catalytic activity in QueDs. This discovery has aroused the interest of many experts and scholars. Yingji Sun et al. found in the inorganic simulation bionic experiment that the reaction activity sequence of complexes containing different transition metals was as follows: Fe^2+^ > Cu^2+^ > Co^2+^ > Ni^2+^ > Zn^2+^ > Mn^2+^ [13]. Recently, through the comparative study of two quercetinases containing manganese and nickel, Sam et al. found that the secondary coordination residues of the active site can regulate the electronic structure of the enzyme-substrate complex through hydrophobic interactions, thus counteracting the effects caused by metal substitution [15]. In theory, Yongjun Liu et al. also discussed the details of the catalytic mechanism of Ni-QueD and Fe-QueD through the QM/MM theoretical research method in which they believed their rate-determining steps were different [21]. In addition, our previous work combined QM/MM calculations and MD simulations to investigate the detailed mechanisms of wild-type Ni-QueD and its Glu76Asp and Glu76Gln mutants, and elucidated the critical roles of the nickel-coordinated Glu76 residue at the active site [22]. Similarly, in this paper, we hope to compare the reaction differences with different active centers of metal ions and describe the specific role and influence of metal ions in enzyme catalysis, so as to achieve better guidance of efficient experimental catalysis by regulating reaction conditions.

**Figure 2:**
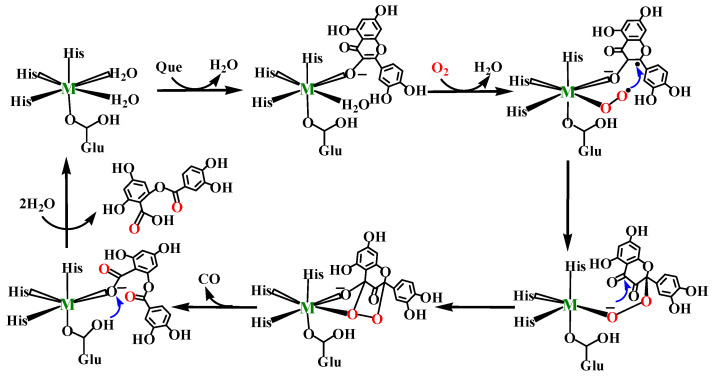
Basic reaction mechanism of metal-containing QueDs.

Based on our previous work, the catalytic reaction cycle involved in the whole reaction of nickel-containing QueDs can be roughly divided into five steps, as shown in Figure 2: (1) The substrate and the quasi-super oxide group coordinated to the metal ion undergoing a biradical reaction to form a peroxide-bridge species in order to achieve the C2–Od bonding step; (2) Bond breaking occurs between the metal ion and the O3 atom previously coordinated to the metal ion by the substrate, thereby adjusting the metal ion to the optimal coordination configuration; (3) The Op–Od bond rotates around the metal ion center to assist in the following ring formation steps; (4) The nucleophilic attack of the negative Op anion on the C4 atom of the substrate to form a five-membered ring step; (5) The fracture of the Op–Od bond leads to ring opening and oxidation of the five-membered ring, releasing CO and its products [22]. However, compared with the nickel ion, the different effects of quercetinases with other metal ions, as the reactive centers on specific reaction steps such as dioxygen activation and ring-opening cleavage or the influences on the reaction rate, require further study and discussion.

Therefore, in order to explore the influence of metal ion on the dioxygen activation, we used the DFT method [23,24,25] to calculate and compare the activation difficulty of dioxygen from the stable triplet state to the active singlet state in three combinations of dioxygen itself, dioxygen with the metal ion, and dioxygen with the substrate under two different conditions of the vacuum environment and the protein environment with the addition of point charge. It was found that compared with the substrate and the protein environment, the metal ion has a greater effect on the process of dioxygen activation. At the same time, the catalytic mechanisms of bacterial QueDs containing six different transition metal ions (Mn^2+^, Fe^2+^, Co^2+^, Ni^2+^, Cu^2+^ and Zn^2+^) were studied and compared by QM/MM calculations [26,27,28]. Our study found that the reactivity sequence of metal ions is Ni^2+^ > Co^2+^ > Zn^2+^ > Mn^2+^ > Fe^2+^ > Cu^2+^, which is basically consistent with the previous experimental results [19,20]. Our results also show that the metal ion only plays the role of Lewis acid in the catalytic reaction and does not play a REDOX role. Furthermore, the coordination tendency of the transition metal ion center also has an important influence on the whole enzyme-catalyzed cycle.

## 2. Results and Discussion

### 2.1. Influencing Factors of Dioxygen Activation

**Figure 3:**
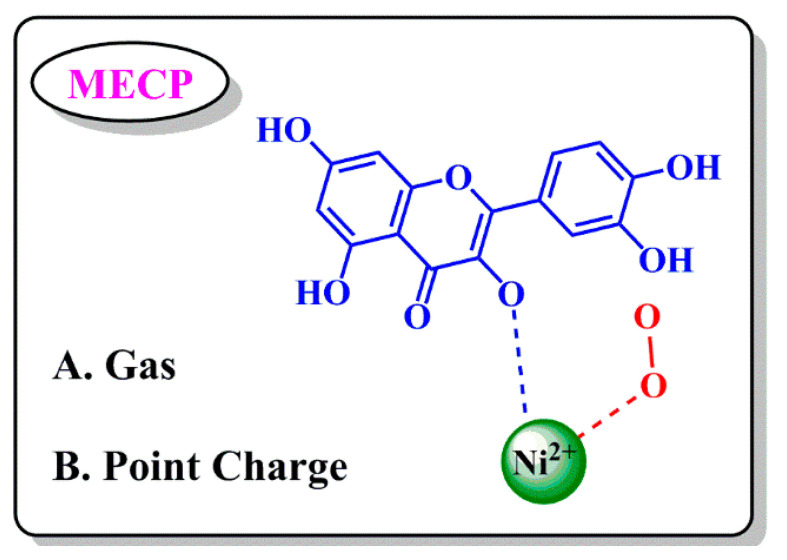
Computational model diagram.

Three main influencing factors were investigated and compared in relation to the dioxygen-activated models of Ni-QueD enzyme: metal ions, substrate, and protein environment. We simulated dioxygen (O_2_), nickel ion and dioxygen (Ni+O_2_), substrate and dioxygen (Que+O_2_) models in vacuum (Gas) and added protein Point Charge (PC), respectively (Figure 3). The activated MECP_(S-T)_ of dioxygen from the stable triplet state to the active singlet state in each model was calculated. The calculated results of the energy of all models in singlet and triplet states and the lowest energy crossing point between the two spin multiplicities are shown in Table 1.

According to the calculation results, we found that the contribution of nickel ion to dioxygen activation (E(Ni+O_2_)-E(O_2_)) is the largest compared with that of dioxygen alone. As a result, the MECP values are reduced by 8.5 kcal/mol and 9.8 kcal/mol in vacuum and adding protein point charge, and the substrate contribution (E(Ni+O_2_)-E(O_2_)) is 5.5 kcal/mol and 6.8 kcal/mol in the above two environments, respectively. Protein environmental contribution to ΔE(Gas-PC) ranges from 1.1 to 2.4 kcal/mol. By comparing the contributions of the three factors to dioxygen activation, we can basically draw the conclusion that metal ion, substrate and protein environment all have certain effects on dioxygen activation, among which metal ion is the most important influencing factor, followed by substrate, and protein environment also has relatively small effects.

Moreover, in order to understand the errors between different calculation methods, three methods (UB3LYP, UCCSD(T) and M062X) were used under the unified basis set to calculate the MECP between the open-shell singlet, triplet and two spin multiplicities of individual dioxygen molecule. As shown in Table S1 in Supporting Information (SI), we found that there were some numerical errors in the results calculated by different methods. In addition, the energy difference between the triplet state and the singlet state of dioxygen measured in the experiment is about 22 kcal/mol [29], which is also different from our calculated results. However, our main concern in this paper is the relative magnitude and general trend of the effects of metal ions, substrates, and protein environments on dioxygen activation, independent of the absolute values produced by different calculation methods. Metal ions are still the most important factors affecting dioxygen activation. Subsequently, we compared the details of the reaction mechanism of six different models activating dioxygen and catalytic cracking quercetin in QueDs enzyme, and explored the differences of the influence of various metal ions on the enzyme-catalyzed reaction.

### 2.2. Electronic and Structural Characteristics of Metal-Containing QueDs Initial Reactants

**Figure 4:**
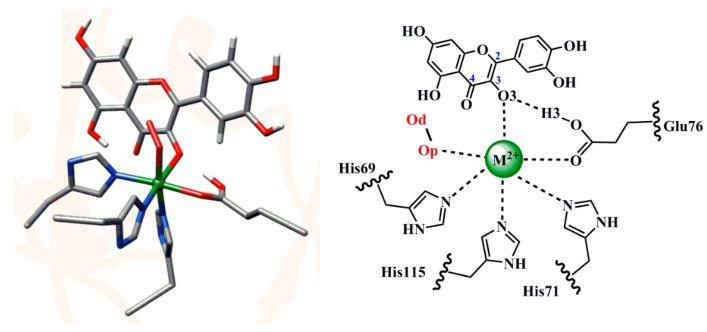
Reactive QM region of QueDs.

Through the combined QM/MM calculation method, we studied the initial reactant structures, and the reaction mechanism of QueDs containing six different metal ions (M^2+^ = Mn^2+^, Fe^2+^, Co^2+^, Ni^2+^, Cu^2+^ and Zn^2+^). The structures of M^2+^-O_2_-Que adducts are shown in Figure 4. In addition, Table S2 shows the electron spin densities, the important structural parameters, and the relative energies of every part of the M^2+^-O_2_-Que adducts.

As shown in Table S2 in the SI among the ground state reactants with the lowest relative energy (shown in blue), the spin densities show that the d-electrons of five metal ions (Mn^2+^, Fe^2+^, Co^2+^, Ni^2+^ and Cu^2+^) present high-spin configurations. The electron spin density of the O_2_ parts of the adducts of Mn^2+^, Fe^2+^, Co^2+^ and Ni^2+^ are close to −2, indicating that there are two spin-down electrons in the molecular orbit of dioxygen, and the relationship with the d-electrons of the corresponding metal ion is antiferromagnetically coupled. However, the electron spin density of the O_2_ parts of the adducts of Cu^2+^ are close to +2, indicating that there are two spin-up electrons in the molecular orbit of dioxygen. From the analysis of the number of single electrons contained in these metal ions, four metal ions mentioned above (Mn^2+^, Fe^2+^, Co^2+^ and Ni^2+^) have more than one single electron, and they form anti-ferromagnetic coupling with the triplet dioxygen to reduce the overall energy. In comparison, the Cu^2+^ has only one single electron and forms a ferromagnetic coupling with the triplet dioxygen. At the same time, we can see that the Op–Od bond length values are between 1.21~1.25 Å in combination with the structural information, indicating that O_2_ is basically not activated at this time. However, in the reaction state shown in red, we note that the electron spin density of the O_2_ moiety is close to +0.5 in the adducts of Mn^2+^, Co^2+^, and Ni^2+^, and their Op–Od bond lengths are larger than or equal to 1.25 Å, indicating that O_2_ in these adducts has undergone a degree of activation and can act as a quasi-superoxide group with reactivity [30]. In addition, we find that the electron spin density of the O_2_ part in the adduct of Cu^2+^ reached 1.20, and its Op–Od bond length is also 1.27 Å, which indicates that O_2_ in the adduct of Cu^2+^ was activated into a superoxide species. Meanwhile, the electron spin density of the O_2_ part in the adduct of Fe^2+^ is just 0.15, which also implies that the catalytic activity of QueD containing divalent iron ion is relatively poor. It is worth noting that the electron spin density of the O_2_ part of the zinc ion without redox ability is also 0.20, indicating that the dioxygen at this time is also slightly activated. In addition, we can find that in all the spin states of all adducts, the O3–H3 bond length is within the range of 1.53~1.68 Å, which indicates that the Glu residues in these adducts have all seized the hydroxyl hydrogen protons of the substrate and formed a relatively stable hydrogen-bond interaction with the substrate. This process is essentially a proton-coupled electron transfer (PCET) process, in which different metal ions show different degrees of promotion. Combined with the data in the table, we can also draw the regularity conclusion about the subsequent reactive spin states. In the reactive state of each adduct containing Mn^2+^, Fe^2+^, Co^2+^, Ni^2+^, Cu^2+^ and Zn^2+^, five divalent transition metal ions (Mn^2+^, Fe^2+^, Co^2+^, Ni^2+^ and Cu^2+^) are in a high-spin state, and Zn^2+^ is always in the closed-shell state. The overall spin multiplicities of the reactants are in descending order from sextet to singlet states.

### 2.3. Comparison of Reaction Mechanism of QueDs Containing Different Metal Ions

We systematically investigated the details of the reaction mechanism of QueDs containing six different transition metal ions. Our calculation results show that the reaction process of all models is basically similar to the reaction mechanism of Ni-QueD described in our previous paper, which is summarized in detail in Figure 2.

**Figure 5:**
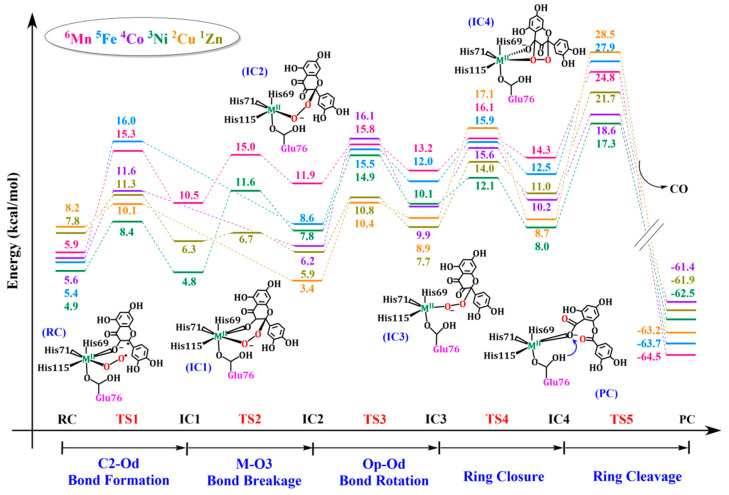
Potential energy surface for the catalysis of metal-containing QueDs.

Figure 5 shows the potential energy surface of QueDs reaction with Mn^2+^, Fe^2+^, Co^2+^, Ni^2+^, Cu^2+^ and Zn^2+^. The overall reactive spin multiplicities of QueDs corresponding to Mn^2+^, Fe^2+^, Co^2+^, Ni^2+^, Cu^2+^ and Zn^2+^ are in the order of sextet quintet, quartet, triplet, doublet and singlet states. As shown in Figure 5, not all metal models underwent the five steps in the basic reaction mechanism, among which only the three metal models with Mn^2+^, Ni^2+^ and Zn^2+^ underwent all five steps, while the other three metal models with Fe^2+^, Co^2+^ and Cu^2+^ went through only four steps without the M–O3 bond-breaking process. As shown in Figure 2, the coordination configuration changes of metal ion centers are involved in this reaction. The models with Fe^2+^, Co^2+^ and Cu^2+^ do not undergo the M–O3 bond-breaking process, indicating that these divalent transition metal ions themselves tend to have a five-coordination configuration, so the M–O3 bond is automatically broken at the first C2–Od bond-forming step to form a relatively stable configuration. However, the models with Mn^2+^, Ni^2+^ and Zn^2+^ tend to have a hexagonal configuration, so they must go through the process of breaking the M–O3 bond to overcome certain reaction energy barrier and form intermediates, which are relatively more suitable for subsequent reactions, and then carry out the Op–Od bond rotation process. These results indicate that the coordination configuration of metal ions also affects the enzymatic reaction to some extent.

**Figure 6:**
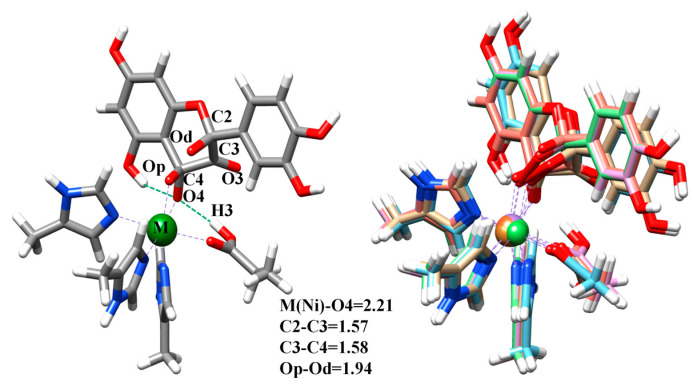
Overlap of QM/MM optimized structures of the TS5 at their reactive states in the metal-containing QueDs. The metal nickel ion is used as an example, and the distances are in Å.

In Figure 5, it is obvious that all models have the same rate-determining step, which is the final ring cleavage step. Additionally, in Figure 6, the structures of the transition state (TS5) of the rate-determining steps are almost completely overlapping without much difference. In addition, by comparing the energy required for the reaction in this step, the reaction activity order of metal ions is as follows: Ni^2+^ > Co^2+^ > Zn^2+^ > Mn^2+^ > Fe^2+^ > Cu^2+^. This result is basically consistent with the order of reactivity Ni^2+^ > Co^2+^ > Mn^2+^ mentioned in the experimental results [19,20]. Furthermore, the details of the reaction mechanism of the model containing Fe^2+^ are basically consistent with the results obtained by the QM/MM method in Yongjun Liu’s research group [21]. However, it is interesting to note that the reaction mechanism of the bacterial QueD containing Cu^2+^ is quite different from the previous result of Saito and Siegbahn et al. [31,32]. Their rate-determining step and the highest reaction energy barrier are different, which also confirms the fact that Cu^2+^ is only found in fungal QueDs but not in bacterial QueDs. As such, the intrinsic differences between bacterial and fungal QueDs are also worth further exploration in the future. At the same time, it should be emphasized that for divalent metal zinc ions without redox ability, the reaction can still proceed relatively smoothly, indicating that metal ions do not play a redox role in the reaction. In addition, in the whole reaction process of all metal models, the oxidation valence state of metal ions did not change and always remained at +2, which can also indicate that the metal ions only act as Lewis acid to stable the substrate anion and the superoxygen and peroxygen groups in the reaction process, and do not play a REDOX role in the reaction. As for the important role of metal ions in dioxygen activation, we think that there are mainly two aspects. On the one hand, partial orbital overlap occurs between metal ions and coordinated dioxygen molecules, which plays a role in activating dioxygen to some extent. On the other hand, during the reaction process, metal ions activate the substrate into a species containing a free radical and, indirectly, further activates dioxygen into another free radical state through the substrate, promoting the smooth occurrence of the subsequent biradical reaction.

## 3. Computational Details

### 3.1. System Preparation

According to the present experimental studies, it is mainly believed that bacterial QueDs derived from the Streptomyces sp. strain FLA can take a variety of different transition metal ions as the reactive center [13,19]. Therefore, all calculations described in this study are based on the X-ray crystal structure of Ni-QueD^FLA^ (PDB code: 5FLJ, resolution: 1.82Å, chain C) [14], including the protein enzyme, the quercetin substrate and dioxygen (ESO_2_), which was constructed in our previously published work [22].

Firstly, we used the PROPKA program [33] as well as the VMD V1.8.6 software (University of Illinois Urbana-Champaign, Champaign, IL, USA) [34] to determine the protonation states of all titratable amino acid residues. Secondly, the missing hydrogen atoms were added by the HBUILD module [35] and optimized by the CHARMM36 force field [36]. After the addition of all hydrogens, the entire system was solvated in TIP3P [37] water sphere in the 16 Å range to filling water molecules in the cavity of the enzyme protein to reach the equilibrium. Finally, sufficient MD simulations were performed under random boundary conditions at 298 K to achieve the energy minimization of the system. To prevent collapse of the coordination environment, metal ions and their coordination residues [30,38] are usually fixed in MD simulations, and detailed procedures can be referred to in the previously published article [22].

### 3.2. QM/MM Calculations

**Figure 7:**
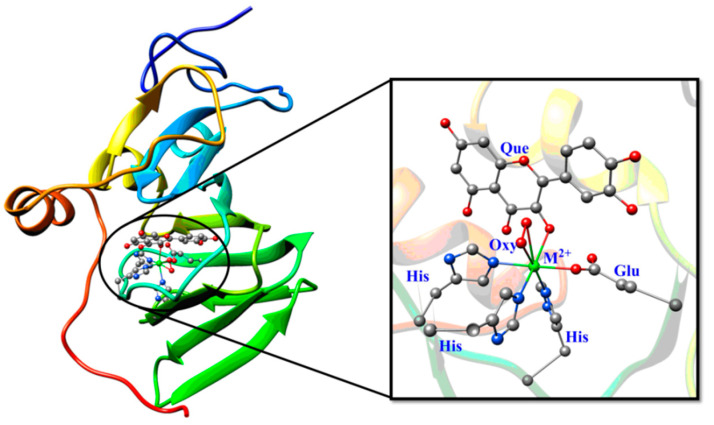
Structural diagrams for QueD (**left**) and its active site (**right**).

Different from the previous simulation structure, in order to compare the effects of different metal ions on the catalytic reactivity of QueDs in the following QM/MM calculations, we replaced five other transition metal ions (manganese, iron, cobalt, copper, zinc) for the nickel ion. As shown in Figure 7, the calculation regions are divided into the MM region (left) treated by molecular mechanics, and the QM region (right) described by quantum mechanics. The QM region contains 81 atoms, including the metal ion, quercetin substrate, dioxygen, and three histidines and a glutamate, whereas the MM region contains the rest of the system. 

All QM/MM calculations are performed by calling the Turbomole V7.1program (Turbomole GmbH, Karlsruhe, Germany) [39] and DL_POLY_4 V5.0.0 program (Daresbury Laboratory, Daresbury, UK) [40] through the ChemShell package [41]. The QM mm boundary problem was treated by the hydrogen link atoms with the charge shift model [42], and the electrostatic interaction between the QM region and the MM region is described by the electron-embedding scheme [43]. The QM region is described by the unrestricted hybrid density functional (UB3LYP) method [44]. The geometric optimization is combined with a basis set (Def2-TZVP for metal ions, Def2-SVP for other atoms [45], labeled as B1). On the basis of configuration optimization, the single point energy calculation for all atoms was performed with the larger basis set Def2-TZVPP [45], denoted as B2.

During the whole study, the CHARMM36 force field [36] was applied to the MM region. The geometric configuration optimization was performed by the DL-FIND optimizer [46] combined with the limited memory BFGS (L-BFGS) method [47] to locate the energy minimum, and employed the Dimer algorithm [48] to search for transition states (TSs). The vibration frequencies were calculated at the theoretical level of UB3LYP/B1, describing each stationary point as a local minimum or TS, and obtaining the zero-point energy (ZPE). All the TSs were optimized and obtained from the highest point of the potential energy surface, where a small increment of 0.02 Å was used to scan the TSs. Furthermore, they were finally determined by analyzing the scanning processes and observing whether the vibration modes of the imaginary frequency TSs correspond to the relevant reaction steps. In all QM calculations, the dispersion correction was performed by the DFT-D3 method [49] to improve the accuracy of B3LYP in describing the weak interactions. The QM/MM energies reported in our work are single point energy combined with ZPE and dispersion correction.

### 3.3. Calculation of MECP

We set up six different scenarios of dioxygen self-activation, dioxygen and nickel ion activation, and dioxygen and substrate activation under two different environments in vacuum and when adding protein point charge. By calculating the MECP of dioxygen from a stable triplet state to an unstable singlet state in these models, we compared and discussed the degree of dioxygen activation in the above six different scenarios. It should be noted that the purpose of this paper is to compare the influences of metal ions, substrate and protein environment on dioxygen activation. Based on the experimental fact that Ni-QueD has the highest catalytic activity, we selected the model containing nickel ion as the research object. As for the calculation of the lowest energy crossing point between the singlet and triplet states of the initial reactants of each model, UB3LYP functional [44] was adopted to perform MECP optimization calculation for the set models through the ORCA program [50]. The Def2-TZVP group is used for nickel ions and the Def2-SVP group for all other atoms [45]. Moreover, we also compared the results calculated by UCCSD(T) [51] and M062X [52] methods to investigate the error between the different calculation methods.

## 4. Conclusions

In this paper, we first analyzed the factors affecting dioxygen activation after the vacuum environment and the added protein-point-charge environment by calculating MECP, and determined that metal ions could have more important implications on dioxygen activation than the substrate organic molecules and protein environment. Subsequently, we systematically compared the electronic structure characteristics and reaction mechanism details of QueDs containing six different transition metals, Mn^2+^, Fe^2+^, Co^2+^, Ni^2+^, Cu^2+^ and Zn^2+^ via a combined QM/MM method. Our calculations show that except for Zn^2+^, which is always in the closed shell state, the d-orbital electrons of the other five metal ions (Mn^2+^, Fe^2+^, Co^2+^, Ni^2+^ and Cu^2+^) are arranged with high spin in their active reaction states and the oxidation valence states of all metal ions during the reaction are always +2. In addition, the speed-determination steps of all model reactions are exactly the same, which is the final ring-opening cleavage step, and the final order of reactivity of metal ions is Ni^2+^ > Co^2+^ > Zn^2+^ > Mn^2+^ > Fe^2+^ > Cu^2+^. Interestingly, the non-redox Zn^2+^ still showed good reactivity, indicating that metal ions do not play a redox role in the catalytic reactions. Furthermore, the coordination configuration tendency of transition metal ion centers also has an important influence on the reaction mechanism.

In conclusion, our calculations suggest that metal ions play important roles in QueDs activation of dioxygen and cleavage of substrate quercetin. On the one hand, the metal ions as Lewis acids, stabilize the substrate anions and subsequent superoxide and peroxy groups in the reaction without exhibiting any redox related effects. On the other hand, the dioxygen and the organic substrate molecules are activated to become free radicals, thus generating the biradical reaction and promoting the subsequent reaction.

## Figures and Tables

**Table 1:** The energy of all models at singlet and triplet states and the MECP of the two spin states.

Species	ΔE(Gas) (kcal/mol)	ΔE(PC) (kcal/mol)	ΔE(Gas-PC) (kcal/mol)
O_2_-Singlet	10.5	10.5	0.0
O_2_-Triplet	0.0	0.0	0.0
O_2_-MECP_(S-T)_	27.0	25.9	1.1
Ni+O_2_-Singlet	0.0	0.0	0.0
Ni+O_2_-Triplet	6.4	6.2	0.2
Ni+O_2_-MECP_(S-T)_	18.5	16.1	2.4
Que+O_2_-Singlet	8.1	7.9	0.2
Que+O_2_-Triplet	0.0	0.0	0.0
Que+O_2_-MECP_(S-T)_	21.5	19.1	2.4

## Supplementary Materials

The following supporting information can be downloaded at: https://www.mdpi.com/article/10.3390/molecules28176238/s1, Table S1: Different functional methods calculate the energy results of dioxygen at singlet and triplet states and the MECP of the two spin states; Table S2: Spin densities, important structural parameters, relative energy of M^2+^-O_2_-Que adducts at different spin states; Coordinates of QM/MM optimized reactants are also listed in detail.
