# Supplementary material for: Unraveling the Pivotal Roles of Various Metal Ion Centers in the Catalysis of Quercetin 2,4-Dioxygenases
Source: Molecules. 2023 Aug 25;28(17):6238. doi: 10.3390/molecules28176238 (PMC10488974; doi:10.3390/molecules28176238)
Supplement: Supplementary file 1 [file molecules-28-06238-s001.zip › molecules-2571995-supplementary.pdf]

Supporting Information

Unraveling the Pivotal Roles of Various Metal Ion Centers in the Catalysis of Quercetin 2,4-Dioxygenases

Xueyuan Yan [1,*], Han Xiao [2], Jinshuai Song [3] and Chunsen Li [2,4,*]

[1] College of Chemistry & Chemical and Environmental Engineering, Weifang University, Weifang 261061, China
[2] State Key Laboratory of Structure of Chemistry, Fujian Institute of Research on the Structure of Matter, Chinese Academy of Sciences, Fuzhou 350002, China
[3] Institute of Green Catalysis, College of Chemistry, Zhengzhou University, Zhengzhou 450001, China; jssong@zzu.edu.cn
[4] Fujian Provincial Key Laboratory of Theoretical and Computational Chemistry, Xiamen University, Xiamen 361005, China
* Correspondence: yanxueyuan@wfu.edu.cn (X.Y.); chunsen.li@fjirsm.ac.cn (C.L.)

Table S1. Different functional methods calculate the energy results of dioxygen at singlet and triplet states and the MECP of the two spin states

O_2	Def2-TZVPP			Def2-SVP		
	E(abs)	E(min)	ΔE	E(abs)	E(min)	ΔE
M062X-R1	-150.27	-150.33	37.0	-150.08	-150.14	37.8
M062X-U1	-150.30	-150.33	14.2	-150.12	-150.14	14.3
M062X-U3	-150.33	-150.33	0.0	-150.14	-150.14	0.0
UCCSD(T)-R1	-149.60	-149.69	53.8	-149.41	-149.49	53.3
UCCSD(T)-U1	-149.66	-149.69	17.6	-149.47	-149.49	17.9
UCCSD(T)-U3	-149.69	-149.69	0.0	-149.49	-149.49	0.0
B3LYP-R1	-150.33	-150.39	38.7	-150.14	-150.20	39.5
B3LYP-U1	-150.37	-150.39	10.1	-150.19	-150.20	10.4
B3LYP-U3	-150.39	-150.39	0.0	-150.20	-150.20	0.0

Table S2. Spin densities, important structural parameters, relative energy of M^{2+}-O_2-Que adducts at different spin states. The ground state information of the reactant is shown in blue, and the reactive state information is displayed in red.

Species	Spin State	ΔE (kcal/mol)	Spin Density			Distance		
			Metal	O_2	Que	d(Op-Od)	d(M-O3)	d(O3-H3)
Mn^{2+}	doublet	16.1	2.19	-1.11	-0.02	1.29	1.96	1.55
	quartet-a	0.0	4.57	-1.52	-0.15	1.25	2.06	1.58
	quartet-b	19.7	2.17	0.87	0.02	1.30	1.93	1.56
	sextet	5.9	4.41	0.58	-0.08	1.27	2.02	1.60
Fe^{2+}	singlet	25.7	0.00	0.00	0.00	1.28	2.22	1.56
	triplet	0.0	3.68	-1.67	-0.15	1.23	2.06	1.59
	quintet	5.4	3.95	0.15	-0.03	1.26	2.02	1.57
Co^{2+}	doublet	0.0	2.70	-1.70	-0.17	1.23	2.05	1.57
	quartet	6.0	2.68	0.37	-0.23	1.25	2.07	1.60
	sextet	3.0	3.00	2.00	0.00	1.22	2.05	1.56
Ni^{2+}	singlet	0.0	1.67	-1.67	-0.22	1.23	2.09	1.56
	triplet	5.1	1.65	0.49	-0.37	1.25	2.15	1.61
	quintet	2.7	1.69	1.78	0.28	1.22	2.07	1.55
Cu^{2+}	doublet	8.2	0.52	1.20	-0.85	1.27	2.70	1.68
	quartet	0.0	0.66	1.89	0.21	1.21	2.01	1.53
Zn^{2+}	singlet	7.8	0.00	0.20	-0.20	1.23	2.49	1.57
	triplet	0.0	0.00	2.00	0.00	1.22	2.04	1.55

Coordinates of QM/MM optimized reactants

Mn-doublet

C -3.692684 -10.135103 2.470094
H -4.129624 -9.917717 3.456287
H -4.512177 -10.094190 1.738880
N -1.822067 -8.485124 3.020901
H -1.842475 -8.609359 4.042778
C -2.639023 -9.138171 2.113710
C -0.954636 -7.709938 2.336410
H -0.191880 -7.073768 2.774580
N -1.169023 -7.831383 1.028209
C -2.218476 -8.716950 0.878528
H -2.598443 -9.004660 -0.094245
C -3.182700 -9.530802 -4.852594
H -3.857010 -8.981244 -5.528070

H -2.277266 -9.820962 -5.407672
N -3.588690 -7.822598 -3.006752
H -4.557841 -7.615473 -3.292086
C -2.756756 -8.686210 -3.695273
C -2.893919 -7.252065 -1.996211
H -3.279842 -6.531349 -1.274261
N -1.645570 -7.713538 -1.992398
C -1.537870 -8.582824 -3.063589
H -0.600075 -9.053823 -3.338588
C 2.868905 -6.280751 -4.445782
H 3.222298 -5.494475 -3.759482
H 3.151302 -7.260105 -4.038095
C 1.343417 -6.173305 -4.587978
H 0.925342 -7.078357 -5.065214
H 1.036360 -5.314936 -5.199469
C 0.659585 -6.060037 -3.258149
O 0.999326 -6.777134 -2.305958
O -0.297746 -5.174314 -3.191028
C 2.977764 -11.631849 1.054756
H 3.359100 -12.438530 0.407491
H 3.851846 -11.060274 1.398065
N 1.083169 -11.226102 -0.583570
H 0.917858 -12.212357 -0.833076
C 2.033655 -10.749856 0.301028
C 0.342088 -10.199262 -1.031916
H -0.489640 -10.321813 -1.718304
N 0.782479 -9.061985 -0.506597
C 1.840008 -9.389186 0.325973
H 2.379747 -8.631945 0.884167
Mn -0.073690 -7.166447 -0.574835
O 1.440531 -6.566423 0.422429
O 1.355454 -6.138778 1.635299
C -3.260511 -4.524050 4.716127
C -3.239535 -4.985084 3.404525
C -2.270911 -4.467977 2.493890
C -1.350018 -3.514267 2.952482
C -1.378761 -3.014827 4.254803
C -2.341615 -3.536838 5.134305
C -2.198757 -4.939053 1.134463
C -0.978215 -4.604259 0.398579
C -0.107483 -3.654458 0.912079
C 1.176815 -3.164097 0.386854
C 1.973584 -2.345047 1.214124
C 3.224374 -1.904485 0.787644
C 3.712596 -2.261026 -0.467776
C 2.929478 -3.071415 -1.319199
C 1.676874 -3.505533 -0.890615
O -0.398262 -3.057224 2.109402
O -3.090494 -5.679438 0.651850
O 3.401279 -3.422365 -2.541409
O 4.916043 -1.880922 -0.963061
O -0.742754 -5.331223 -0.700016
O -2.428113 -3.147891 6.414672
O -4.099090 -5.916204 2.982999
H -3.990559 -4.915212 5.425428
H -0.637626 -2.285600 4.584857
H 1.624635 -2.059343 2.204027
H 3.833498 -1.276276 1.443999
H 1.096472 -4.122487 -1.562858
H 4.337383 -3.154417 -2.627315
H 5.437562 -1.427016 -0.289100
H -1.888326 -2.334138 6.589124
H -3.925590 -6.020630 1.998695
H -0.647436 -5.125936 -2.236844
H -3.311200 -11.154079 2.535102
H -3.697387 -10.438239 -4.536806
H 3.330105 -6.163694 -5.426424
H 2.506238 -12.147628 1.891239

Mn-quartet-a

C -3.774727 -10.250889 2.526195
H -4.234805 -10.074277 3.509848
H -4.582752 -10.217454 1.782066
N -1.939866 -8.595226 3.151106
H -1.950259 -8.760228 4.168706
C -2.750194 -9.210429 2.215352
C -1.087519 -7.773776 2.496545
H -0.343205 -7.156382 2.992071
N -1.304263 -7.824827 1.190172
C -2.340702 -8.711354 1.001688
H -2.722760 -8.949207 0.013452
C -3.183530 -9.561330 -4.848188
H -3.846611 -9.022633 -5.543470
H -2.270181 -9.861033 -5.384957
N -3.612448 -7.805676 -3.052071
H -4.582162 -7.611048 -3.347047
C -2.772682 -8.690272 -3.704979

C	-2.925099 -7.194531 -2.061633	
H	-3.316688 -6.444011 -1.375139	
N	-1.674006 -7.647441 -2.035993	
C	-1.558373 -8.560193 -3.069878	
H	-0.615064 -9.034505 -3.320938	
C	2.906378 -6.199953 -4.554211	
H	3.261424 -5.402362 -3.880946	
H	3.181296 -7.170162 -4.119562	
C	1.382296 -6.090712 -4.699335	
H	0.962074 -7.001759 -5.162299	
H	1.074764 -5.239233 -5.319631	
C	0.729175 -5.967406 -3.354334	
O	1.075320 -6.677876 -2.409650	
O	-0.214034 -5.057278 -3.274391	
C	2.985191 -11.656301 1.097109	
H	3.368517 -12.461953 0.449463	
H	3.859496 -11.088729 1.446217	
N	1.096671 -11.242309 -0.543116	
H	0.924359 -12.227611 -0.788548	
C	2.050490 -10.769417 0.338173	
C	0.370967 -10.204744 -0.999607	
H	-0.461583 -10.321523 -1.687132	
N	0.819306 -9.068460 -0.484884	
C	1.868114 -9.404976 0.349694	
H	2.420833 -8.653401 0.905221	
Mn	-0.146000 -7.109924 -0.588993	
O	1.404028 -6.533802 0.706595	
O	1.176091 -5.854512 1.727428	
C	-3.185333 -4.496580 4.667663	
C	-3.153225 -4.922998 3.344428	
C	-2.185928 -4.371978 2.450898	
C	-1.271713 -3.424981 2.940403	
C	-1.313361 -2.961054 4.254897	
C	-2.278225 -3.512244 5.115333	
C	-2.114380 -4.796991 1.077962	
C	-0.891803 -4.431190 0.346923	
C	-0.016959 -3.500273 0.906000	
C	1.275349 -3.027707 0.394900	
C	2.082135 -2.225088 1.230163	
C	3.346801 -1.816150 0.813435	
C	3.840496 -2.187404 -0.436607	
C	3.048740 -2.983041 -1.295805	
C	1.781217 -3.385624 -0.878551	
O	-0.310971 -2.941690 2.119826	
O	-2.992514 -5.531826 0.567814	
O	3.519031 -3.348408 -2.511987	
O	5.058532 -1.837776 -0.915969	
O	-0.640147 -5.117009 -0.748686	
O	-2.375070 -3.153804 6.403734	
O	-4.002574 -5.850433 2.896296	
H	-3.915652 -4.912490 5.362443	
H	-0.581215 -2.234452 4.609479	
H	1.729131 -1.930329 2.216525	
H	3.964098 -1.198773 1.472600	
H	1.190430 -3.990565 -1.554460	
H	4.462102 -3.105266 -2.604898	
H	5.556712 -1.337845 -0.256885	
H	-1.849054 -2.334778 6.595938	
H	-3.824254 -5.932618 1.911727	
H	-0.539689 -4.987054 -2.320890	
H	-3.355984 -11.255845 2.578972	
H	-3.701115 -10.464612 -4.525304	
H	3.375562 -6.110346 -5.533957	
H	2.507623 -12.174772 1.928484	

Mn-quartet-b

C	-3.697692 -10.144969 2.476789	
H	-4.128464 -9.928092 3.465818	
H	-4.520978 -10.102070 1.750030	
N	-1.805293 -8.515562 3.013715	
H	-1.816583 -8.641861 4.035456	
C	-2.645080 -9.149221 2.114280	
C	-0.937234 -7.746792 2.322224	
H	-0.161501 -7.120359 2.753090	
N	-1.177029 -7.851796 1.017250	
C	-2.241808 -8.718963 0.875979	
H	-2.642269 -8.992420 -0.092616	
C	-3.186094 -9.534251 -4.854283	
H	-3.859828 -8.984558 -5.530189	
H	-2.280149 -9.824120 -5.408745	
N	-3.591182 -7.825684 -3.007724	
H	-4.559990 -7.616223 -3.292439	
C	-2.760993 -8.689741 -3.696301	
C	-2.895683 -7.256948 -1.995676	
H	-3.280760 -6.535980 -1.273620	
N	-1.648704 -7.721984 -1.991559	
C	-1.541993 -8.590574 -3.063247	

H	-0.606016 -9.065922 -3.336422
C	2.870374 -6.288262 -4.446189
H	3.218707 -5.499735 -3.759876
H	3.159895 -7.264881 -4.037400
C	1.343860 -6.195079 -4.587067
H	0.934277 -7.106066 -5.060295
H	1.027187 -5.340159 -5.198416
C	0.663095 -6.087063 -3.255697
O	1.007196 -6.806148 -2.305980
O	-0.294875 -5.203185 -3.183195
C	2.976170 -11.631353 1.053574
H	3.358622 -12.438314 0.407217
H	3.849205 -11.057374 1.395542
N	1.073700 -11.228098 -0.577371
H	0.903908 -12.214588 -0.821326
C	2.030963 -10.750590 0.299484
C	0.332959 -10.199988 -1.024263
H	-0.505770 -10.322703 -1.701837
N	0.781033 -9.062050 -0.506385
C	1.842846 -9.389056 0.320060
H	2.386209 -8.627662 0.868979
Mn	-0.086840 -7.169209 -0.572630
O	1.449974 -6.611236 0.479707
O	1.292693 -6.053660 1.639728
C	-3.263665 -4.524959 4.718489
C	-3.240471 -4.988183 3.407668
C	-2.274823 -4.467564 2.496176
C	-1.357171 -3.510338 2.953943
C	-1.387899 -3.009085 4.255132
C	-2.348098 -3.534471 5.135923
C	-2.197308 -4.944313 1.139114
C	-0.975697 -4.608187 0.409127
C	-0.111448 -3.650026 0.916975
C	1.171080 -3.159177 0.389721
C	1.969499 -2.342323 1.217235
C	3.221019 -1.904472 0.790501
C	3.708278 -2.262262 -0.465050
C	2.923442 -3.071011 -1.316470
C	1.669635 -3.501613 -0.888090
O	-0.407607 -3.049050 2.109167
O	-3.085059 -5.689502 0.655862
O	3.394105 -3.422019 -2.539178
O	4.912742 -1.886016 -0.960339
O	-0.720752 -5.348050 -0.678958
O	-2.434201 -3.145192 6.416211
O	-4.095695 -5.924404 2.988353
H	-3.992350 -4.919162 5.427508
H	-0.648783 -2.277571 4.584709
H	1.621920 -2.057647 2.207979
H	3.831664 -1.278061 1.447044
H	1.087682 -4.116537 -1.561171
H	4.331216 -3.157417 -2.624314
H	5.437097 -1.435958 -0.285879
H	-1.894579 -2.331143 6.589896
H	-3.922085 -6.031302 2.004809
H	-0.640866 -5.156741 -2.229971
H	-3.315739 -11.163906 2.539607
H	-3.700042 -10.442081 -4.538421
H	3.330526 -6.168453 -5.426991
H	2.506034 -12.146586 1.891175

Mn-sextet

C	-3.787172 -10.266136 2.533806
H	-4.242685 -10.089979 3.519679
H	-4.599007 -10.236824 1.793717
N	-1.955612 -8.602474 3.147368
H	-1.961313 -8.766206 4.165058
C	-2.767517 -9.222807 2.216331
C	-1.108065 -7.779749 2.487615
H	-0.360547 -7.157394 2.971144
N	-1.330404 -7.834531 1.182093
C	-2.364009 -8.725514 0.999930
H	-2.749213 -8.969716 0.014497
C	-3.175257 -9.547508 -4.835097
H	-3.839045 -9.005230 -5.526952
H	-2.262372 -9.844374 -5.374238
N	-3.605239 -7.806866 -3.024703
H	-4.575676 -7.613176 -3.317381
C	-2.763568 -8.682646 -3.687588
C	-2.918810 -7.200101 -2.031982
H	-3.311041 -6.456904 -1.337692
N	-1.665119 -7.648388 -2.014370
C	-1.547618 -8.552551 -3.056416
H	-0.602904 -9.019806 -3.314854
C	2.906285 -6.202193 -4.564303
H	3.258236 -5.403799 -3.890465
H	3.181528 -7.172471 -4.130017

C	1.382201 -6.096511 -4.713738
H	0.967332 -7.005308 -5.185757
H	1.074659 -5.241694 -5.329328
C	0.724526 -5.986247 -3.370204
O	1.062791 -6.707447 -2.432070
O	-0.215494 -5.071465 -3.283685
C	2.969481 -11.626849 1.085824
H	3.357329 -12.426244 0.433124
H	3.840355 -11.053005 1.433447
N	1.065149 -11.225525 -0.539292
H	0.899397 -12.212223 -0.784058
C	2.023338 -10.745255 0.334070
C	0.328908 -10.195297 -0.992250
H	-0.508104 -10.319566 -1.672695
N	0.776671 -9.055048 -0.483799
C	1.834647 -9.381919 0.344375
H	2.385642 -8.622545 0.890554
Mn	-0.147921 -7.113631 -0.588783
O	1.408251 -6.520635 0.613953
O	1.133732 -5.924641 1.699500
C	-3.193565 -4.492755 4.661063
C	-3.162001 -4.925312 3.339815
C	-2.189252 -4.385045 2.446372
C	-1.268876 -3.443601 2.933783
C	-1.309501 -2.973092 4.245631
C	-2.280460 -3.513323 5.106617
C	-2.116180 -4.816905 1.075909
C	-0.888308 -4.465374 0.349843
C	-0.009583 -3.534294 0.902339
C	1.279915 -3.058978 0.387886
C	2.081509 -2.247544 1.219775
C	3.341759 -1.829267 0.800499
C	3.836998 -2.201719 -0.448730
C	3.051046 -3.007407 -1.304028
C	1.787533 -3.418795 -0.883983
O	-0.305061 -2.968380 2.110770
O	-3.002133 -5.541768 0.565223
O	3.523166 -3.372674 -2.519625
O	5.051269 -1.843562 -0.930269
O	-0.635910 -5.159926 -0.744218
O	-2.378328 -3.148118 6.392887
O	-4.017677 -5.848900 2.895568
H	-3.928949 -4.900357 5.355332
H	-0.573016 -2.250335 4.598881
H	1.727524 -1.953085 2.205728
H	3.954748 -1.204771 1.456953
H	1.202732 -4.033191 -1.556340
H	4.464713 -3.123857 -2.613446
H	5.547291 -1.339948 -0.272265
H	-1.848776 -2.331071 6.583875
H	-3.840180 -5.937956 1.912328
H	-0.541585 -5.010596 -2.332231
H	-3.364310 -11.269418 2.585551
H	-3.692851 -10.452285 -4.516439
H	3.377746 -6.110959 -5.542806
H	2.501652 -12.153467 1.917615

Fe-singlet

C	-3.755302 -10.203085 2.452918
H	-4.194739 -9.988523 3.438785
H	-4.576644 -10.185426 1.723141
N	-1.898647 -8.540367 2.987039
H	-1.903566 -8.665458 4.008207
C	-2.722993 -9.187572 2.086534
C	-1.043937 -7.754558 2.292643
H	-0.288199 -7.117751 2.738290
N	-1.275430 -7.859785 0.987524
C	-2.320387 -8.749901 0.848030
H	-2.709132 -9.038585 -0.121209
C	-3.166250 -9.505783 -4.829764
H	-3.841414 -8.951737 -5.500989
H	-2.260127 -9.789917 -5.387025
N	-3.567251 -7.802380 -2.974812
H	-4.535037 -7.589454 -3.257675
C	-2.740233 -8.667678 -3.667257
C	-2.867449 -7.240621 -1.961034
H	-3.248817 -6.516481 -1.241525
N	-1.622359 -7.713405 -1.956743
C	-1.520965 -8.574754 -3.035614
H	-0.586064 -9.049829 -3.311717
C	2.892124 -6.316708 -4.443059
H	3.225661 -5.516663 -3.762518
H	3.204152 -7.284528 -4.030414
C	1.364183 -6.258014 -4.585163
H	0.977673 -7.188223 -5.038887
H	1.034742 -5.423284 -5.217757
C	0.674831 -6.127835 -3.257299

O 0.988070 -6.837662 -2.297530
O -0.268418 -5.218362 -3.225428
C 2.953042 -11.555941 1.037740
H 3.350660 -12.343694 0.376807
H 3.815949 -10.969169 1.384399
N 1.040213 -11.195755 -0.579616
H 0.899857 -12.186596 -0.821783
C 1.981600 -10.691424 0.296319
C 0.262361 -10.189706 -1.020636
H -0.574969 -10.338204 -1.693699
N 0.672658 -9.040007 -0.503958
C 1.746305 -9.334779 0.319167
H 2.269093 -8.558872 0.869816
Fe -0.155603 -7.236865 -0.588275
O 1.137387 -6.404379 0.378502
O 0.842816 -5.805597 1.470089
C -3.163958 -4.549253 4.784371
C -3.164279 -4.993264 3.466608
C -2.199187 -4.478887 2.549739
C -1.261721 -3.539001 3.007663
C -1.273342 -3.052683 4.312417
C -2.236093 -3.571316 5.198724
C -2.185978 -4.894317 1.175708
C -0.991726 -4.523221 0.375664
C -0.064847 -3.602680 0.930592
C 1.177070 -3.080350 0.362627
C 1.979248 -2.240396 1.167895
C 3.223546 -1.810789 0.720714
C 3.701496 -2.198445 -0.531577
C 2.911173 -3.022338 -1.363276
C 1.660910 -3.447703 -0.916502
O -0.314927 -3.070862 2.159099
O -3.096433 -5.595955 0.686218
O 3.373804 -3.396803 -2.580168
O 4.906436 -1.840717 -1.033703
O -0.844813 -5.132896 -0.748431
O -2.310081 -3.183467 6.478622
O -4.047198 -5.899308 3.044523
H -3.894033 -4.937003 5.495156
H -0.528566 -2.327452 4.642197
H 1.640257 -1.941510 2.157492
H 3.841896 -1.170890 1.356724
H 1.071791 -4.080462 -1.567157
H 4.311984 -3.139642 -2.677552
H 5.424638 -1.356302 -0.378357
H -1.793104 -2.352194 6.642922
H -3.899935 -5.991977 2.057343
H -0.628931 -5.120856 -2.290446
H -3.349493 -11.212065 2.526024
H -3.679069 -10.417535 -4.523512
H 3.346215 -6.187075 -5.425436
H 2.499869 -12.097210 1.868239

Fe-triplet

C -3.767283 -10.237409 2.516694
H -4.222111 -10.054329 3.501586
H -4.577868 -10.203491 1.775390
N -1.915613 -8.591812 3.121103
H -1.924524 -8.749137 4.139676
C -2.738285 -9.204922 2.194557
C -1.059018 -7.784367 2.454572
H -0.305980 -7.173233 2.944117
N -1.285238 -7.841738 1.149395
C -2.332305 -8.718540 0.974949
H -2.721363 -8.960591 -0.009160
C -3.192295 -9.569690 -4.858375
H -3.853633 -9.035500 -5.558815
H -2.276343 -9.869469 -5.390701
N -3.637812 -7.823449 -3.057523
H -4.606733 -7.631128 -3.356574
C -2.788846 -8.695093 -3.715291
C -2.956752 -7.215730 -2.059709
H -3.356961 -6.473190 -1.370455
N -1.702400 -7.655758 -2.034968
C -1.576787 -8.556723 -3.077089
H -0.628775 -9.021187 -3.329118
C 2.893469 -6.220302 -4.526834
H 3.242444 -5.419736 -3.853863
H 3.176356 -7.189070 -4.094161
C 1.368085 -6.122711 -4.673141
H 0.955296 -7.037762 -5.134449
H 1.058088 -5.274741 -5.297271
C 0.709753 -5.994385 -3.330224
O 1.035185 -6.711113 -2.384858
O -0.217107 -5.064773 -3.254805
C 2.983161 -11.641810 1.096419
H 3.368812 -12.445152 0.447348

H	3.856048 -11.074036 1.448818
N	1.103694 -11.226379 -0.553113
H	0.936334 -12.211578 -0.802627
C	2.047074 -10.754365 0.339807
C	0.375422 -10.189308 -1.007446
H	-0.451293 -10.304214 -1.702202
N	0.811309 -9.054444 -0.479537
C	1.855652 -9.391120 0.360008
H	2.400452 -8.641763 0.926004
Fe	-0.188312 -7.151454 -0.605869
O	1.434592 -6.482830 0.745394
O	1.199111 -5.811109 1.749939
C	-3.176232 -4.489415 4.672728
C	-3.137995 -4.922527 3.351976
C	-2.164347 -4.378557 2.460842
C	-1.254862 -3.425694 2.948517
C	-1.304498 -2.953624 4.259804
C	-2.272761 -3.501481 5.119044
C	-2.086945 -4.812923 1.090983
C	-0.856723 -4.454679 0.364123
C	0.009275 -3.509695 0.919976
C	1.299015 -3.033918 0.406785
C	2.102137 -2.223422 1.238407
C	3.364928 -1.811089 0.819721
C	3.860281 -2.185555 -0.428935
C	3.072676 -2.990180 -1.283998
C	1.807274 -3.397632 -0.864472
O	-0.292444 -2.943466 2.128682
O	-2.965295 -5.544629 0.580173
O	3.542223 -3.357164 -2.499402
O	5.076421 -1.832069 -0.909519
O	-0.594263 -5.138152 -0.724055
O	-2.375341 -3.137446 6.405164
O	-3.986871 -5.850996 2.905349
H	-3.910026 -4.902111 5.365646
H	-0.575988 -2.223361 4.614195
H	1.746755 -1.924035 2.222544
H	3.979792 -1.188685 1.476417
H	1.219867 -4.011322 -1.535855
H	4.483563 -3.108674 -2.597910
H	5.571121 -1.324223 -0.253740
H	-1.850838 -2.317105 6.596535
H	-3.808281 -5.936314 1.921350
H	-0.539389 -4.995463 -2.301935
H	-3.352598 -11.244014 2.570073
H	-3.708970 -10.473061 -4.534286
H	3.361857 -6.121835 -5.506111
H	2.505425 -12.163573 1.925634

Fe-quintet

C	-3.765120 -10.224965 2.502766
H	-4.204931 -10.032325 3.492712
H	-4.586743 -10.199035 1.773484
N	-1.906032 -8.572641 3.065184
H	-1.906538 -8.718910 4.084937
C	-2.741555 -9.195476 2.155168
C	-1.052456 -7.778467 2.381100
H	-0.288799 -7.158593 2.843904
N	-1.296974 -7.853861 1.078433
C	-2.348784 -8.727297 0.924373
H	-2.747155 -8.983649 -0.051852
C	-3.180617 -9.543347 -4.838928
H	-3.849418 -8.997783 -5.523353
H	-2.271495 -9.836933 -5.386198
N	-3.593780 -7.807976 -3.018776
H	-4.563027 -7.606656 -3.307966
C	-2.760705 -8.685181 -3.688921
C	-2.896266 -7.211532 -2.024169
H	-3.281939 -6.470293 -1.324107
N	-1.646703 -7.667527 -2.012162
C	-1.541549 -8.565026 -3.059984
H	-0.602108 -9.038617 -3.325878
C	2.890454 -6.234947 -4.505587
H	3.240014 -5.436500 -3.830669
H	3.176753 -7.205505 -4.079523
C	1.363799 -6.142767 -4.647270
H	0.954693 -7.061233 -5.105445
H	1.046356 -5.297720 -5.271592
C	0.705340 -6.018767 -3.305421
O	1.045285 -6.738205 -2.361182
O	-0.229131 -5.105212 -3.221219
C	2.999025 -11.660516 1.087527
H	3.380735 -12.472416 0.446774
H	3.874635 -11.096133 1.438390
N	1.116172 -11.241433 -0.559719
H	0.934637 -12.226661 -0.800244
C	2.073881 -10.771499 0.319950

C	0.399598 -10.201867 -1.022944
H	-0.435399 -10.314247 -1.707885
N	0.856322 -9.067067 -0.511747
C	1.903074 -9.406434 0.324851
H	2.455232 -8.656032 0.881799
Fe	-0.088012 -7.131628 -0.571923
O	1.271055 -6.637380 0.780523
O	1.217648 -5.783619 1.707360
C	-3.179722 -4.487757 4.696989
C	-3.157030 -4.920226 3.375845
C	-2.188094 -4.382049 2.477105
C	-1.267026 -3.437044 2.956537
C	-1.301130 -2.964747 4.268035
C	-2.264417 -3.507402 5.135996
C	-2.118490 -4.822943 1.109150
C	-0.889104 -4.479578 0.381771
C	-0.016563 -3.535842 0.920218
C	1.266611 -3.049769 0.394852
C	2.063268 -2.224569 1.215816
C	3.321316 -1.804120 0.790121
C	3.816989 -2.186239 -0.455406
C	3.034033 -3.004304 -1.300791
C	1.773846 -3.419453 -0.873408
O	-0.310683 -2.961196 2.125769
O	-3.007411 -5.548226 0.604714
O	3.506612 -3.381517 -2.513078
O	5.030752 -1.829140 -0.941137
O	-0.635257 -5.193769 -0.701827
O	-2.352632 -3.144628 6.423648
O	-4.016255 -5.842669 2.935796
H	-3.909772 -4.895666 5.396544
H	-0.564176 -2.239935 4.615972
H	1.710049 -1.923128 2.199903
H	3.932656 -1.171228 1.439993
H	1.192576 -4.045977 -1.537540
H	4.447007 -3.129117 -2.610090
H	5.527367 -1.322964 -0.285645
H	-1.828199 -2.323014 6.609739
H	-3.845036 -5.930101 1.950699
H	-0.555462 -5.040842 -2.265153
H	-3.350270 -11.231321 2.559455
H	-3.696543 -10.450336 -4.523876
H	3.354655 -6.132832 -5.486483
H	2.514136 -12.170906 1.919664

Co-doublet

C	-3.763829 -10.221839 2.503755
H	-4.219115 -10.038164 3.488405
H	-4.574108 -10.189294 1.762079
N	-1.903943 -8.584193 3.103978
H	-1.906648 -8.745046 4.121988
C	-2.734542 -9.189891 2.180674
C	-1.047574 -7.777485 2.436279
H	-0.289359 -7.174513 2.927016
N	-1.283970 -7.825841 1.132228
C	-2.336106 -8.697944 0.960768
H	-2.731686 -8.936102 -0.021297
C	-3.196236 -9.561856 -4.867415
H	-3.861439 -9.026215 -5.562967
H	-2.282299 -9.859759 -5.404294
N	-3.632113 -7.818879 -3.061916
H	-4.600138 -7.621138 -3.359419
C	-2.788674 -8.692831 -3.721777
C	-2.948299 -7.221497 -2.058694
H	-3.345154 -6.481062 -1.365351
N	-1.698173 -7.672560 -2.033428
C	-1.577121 -8.568045 -3.079788
H	-0.633503 -9.039451 -3.333923
C	2.883960 -6.250390 -4.505831
H	3.228731 -5.451617 -3.828695
H	3.174427 -7.219663 -4.079638
C	1.357245 -6.163283 -4.650220
H	0.951438 -7.078046 -5.118106
H	1.042311 -5.311747 -5.267178
C	0.696674 -6.048805 -3.305793
O	1.017321 -6.779338 -2.369165
O	-0.224960 -5.115697 -3.222333
C	2.991244 -11.623468 1.087572
H	3.375602 -12.427543 0.438544
H	3.864940 -11.057564 1.441236
N	1.119893 -11.210425 -0.569963
H	0.954788 -12.196049 -0.817593
C	2.056258 -10.736254 0.328392
C	0.390954 -10.174541 -1.027659
H	-0.434074 -10.293443 -1.723579
N	0.818662 -9.039099 -0.494995
C	1.858455 -9.373646 0.350915

H 2.397590 -8.624535 0.922065
Co -0.216842 -7.198698 -0.607092
O 1.416540 -6.498085 0.813974
O 1.188148 -5.781660 1.784122
C -3.172703 -4.501786 4.685309
C -3.141930 -4.932713 3.363800
C -2.168773 -4.391856 2.470550
C -1.256160 -3.440161 2.954616
C -1.298839 -2.969184 4.266545
C -2.264835 -3.516827 5.129180
C -2.092991 -4.831408 1.102427
C -0.855110 -4.490131 0.377858
C 0.006646 -3.533485 0.925270
C 1.292052 -3.054674 0.406009
C 2.085721 -2.221542 1.224415
C 3.343656 -1.801932 0.798800
C 3.841825 -2.189389 -0.444750
C 3.063377 -3.016431 -1.287055
C 1.803038 -3.432185 -0.860349
O -0.297143 -2.958924 2.129603
O -2.979649 -5.551365 0.590556
O 3.538576 -3.398391 -2.495150
O 5.053297 -1.829384 -0.931500
O -0.592607 -5.185030 -0.698773
O -2.361950 -3.154982 6.416285
O -3.996438 -5.856717 2.918834
H -3.904500 -4.913761 5.380699
H -0.567529 -2.240220 4.618575
H 1.726575 -1.910858 2.203683
H 3.952246 -1.163329 1.445637
H 1.221975 -4.063117 -1.521388
H 4.476851 -3.139535 -2.597932
H 5.541884 -1.303722 -0.285285
H -1.837498 -2.334430 6.606794
H -3.821885 -5.941885 1.934339
H -0.544948 -5.053697 -2.267328
H -3.348376 -11.228096 2.557717
H -3.710822 -10.466061 -4.542329
H 3.350435 -6.141488 -5.484916
H 2.510503 -12.144759 1.915347

Co-quartet

C -3.769559 -10.228608 2.503965
H -4.224741 -10.045462 3.488838
H -4.580702 -10.199637 1.763065
N -1.909652 -8.588490 3.097598
H -1.907957 -8.751030 4.115135
C -2.745290 -9.192950 2.177500
C -1.057190 -7.780050 2.426511
H -0.296479 -7.172420 2.907171
N -1.300970 -7.826883 1.123808
C -2.352760 -8.699379 0.956075
H -2.751985 -8.937284 -0.024678
C -3.185527 -9.554382 -4.858829
H -3.849402 -9.018963 -5.555984
H -2.270855 -9.852547 -5.394271
N -3.623410 -7.811378 -3.052404
H -4.592037 -7.615356 -3.348287
C -2.779080 -8.683609 -3.713504
C -2.937918 -7.211547 -2.051185
H -3.337027 -6.471565 -1.358991
N -1.686893 -7.659426 -2.027069
C -1.566334 -8.555273 -3.073365
H -0.621002 -9.022612 -3.328872
C 2.885507 -6.252100 -4.514071
H 3.228620 -5.454727 -3.834608
H 3.174908 -7.221984 -4.088657
C 1.359234 -6.166408 -4.660764
H 0.953546 -7.083475 -5.124340
H 1.041393 -5.319182 -5.282187
C 0.696492 -6.046489 -3.318515
O 1.023434 -6.759037 -2.372334
O -0.239274 -5.123072 -3.249591
C 2.987855 -11.612233 1.086759
H 3.376705 -12.413462 0.436875
H 3.859211 -11.044108 1.442422
N 1.115249 -11.206318 -0.568912
H 0.950306 -12.192747 -0.812876
C 2.052055 -10.727343 0.327174
C 0.386020 -10.172340 -1.030320
H -0.437982 -10.292881 -1.726703
N 0.813278 -9.035441 -0.501629
C 1.853282 -9.364767 0.345379
H 2.389504 -8.609067 0.911234
Co -0.196066 -7.182115 -0.592742
O 1.347333 -6.514435 0.769961
O 1.084989 -5.761208 1.727722

C	-3.164375	-4.501821	4.694588
C	-3.136790	-4.930401	3.371745
C	-2.159861	-4.395941	2.478820
C	-1.237537	-3.455232	2.965408
C	-1.277714	-2.984940	4.276133
C	-2.249647	-3.524656	5.139119
C	-2.093530	-4.826532	1.108696
C	-0.854834	-4.484970	0.376033
C	0.022058	-3.535568	0.931977
C	1.297763	-3.052618	0.403800
C	2.092611	-2.215582	1.219258
C	3.347077	-1.794485	0.789370
C	3.842701	-2.186608	-0.454592
C	3.063477	-3.017223	-1.294219
C	1.806384	-3.435465	-0.862732
O	-0.274078	-2.976231	2.140475
O	-2.986300	-5.532126	0.592205
O	3.536178	-3.399117	-2.502405
O	5.051528	-1.827010	-0.943296
O	-0.606858	-5.160192	-0.705037
O	-2.345615	-3.159281	6.424527
O	-4.000773	-5.843880	2.925651
H	-3.899987	-4.907783	5.389513
H	-0.542263	-2.261190	4.629262
H	1.737104	-1.906800	2.200353
H	3.956909	-1.152566	1.431608
H	1.227251	-4.075398	-1.516868
H	4.475314	-3.143785	-2.607597
H	5.544010	-1.303966	-0.297658
H	-1.822518	-2.337148	6.613588
H	-3.830589	-5.930140	1.941662
H	-0.566329	-5.058751	-2.303350
H	-3.351420	-11.233699	2.558912
H	-3.701297	-10.459094	-4.537047
H	3.353357	-6.142423	-5.492414
H	2.509969	-12.138038	1.913330

Co-sextet

C	-3.778938	-10.249015	2.515763
H	-4.228511	-10.068561	3.503336
H	-4.593615	-10.217730	1.778949
N	-1.926171	-8.599155	3.103796
H	-1.928578	-8.750423	4.123402
C	-2.755334	-9.214166	2.186444
C	-1.070073	-7.798268	2.428801
H	-0.319190	-7.184457	2.916541
N	-1.304596	-7.861049	1.125086
C	-2.356205	-8.735276	0.961598
H	-2.753092	-8.982521	-0.018184
C	-3.229315	-9.595185	-4.888356
H	-3.892359	-9.065039	-5.589698
H	-2.312597	-9.893483	-5.420376
N	-3.674308	-7.845493	-3.091360
H	-4.640873	-7.646671	-3.394845
C	-2.827712	-8.719276	-3.747005
C	-2.995921	-7.245307	-2.087485
H	-3.395247	-6.502342	-1.398661
N	-1.744752	-7.692804	-2.060838
C	-1.618015	-8.590691	-3.103883
H	-0.671107	-9.056829	-3.355765
C	2.881469	-6.239017	-4.498988
H	3.225336	-5.437527	-3.824042
H	3.173646	-7.207000	-4.070585
C	1.353696	-6.161045	-4.642784
H	0.957946	-7.072874	-5.123682
H	1.034081	-5.301298	-5.245560
C	0.696584	-6.072324	-3.294652
O	0.982194	-6.856217	-2.390318
O	-0.179060	-5.101099	-3.168559
C	2.993159	-11.626505	1.107375
H	3.387419	-12.427573	0.460891
H	3.861134	-11.055539	1.466813
N	1.147979	-11.223211	-0.580496
H	0.994876	-12.208915	-0.836284
C	2.058391	-10.745372	0.341637
C	0.417834	-10.192087	-1.047006
H	-0.385627	-10.314103	-1.767440
N	0.818847	-9.056150	-0.494867
C	1.842899	-9.385404	0.372129
H	2.358762	-8.635868	0.963374
Co	-0.280665	-7.264438	-0.635906
O	1.522354	-6.445317	1.044567
O	1.251878	-5.720531	1.983855
C	-3.171727	-4.475296	4.656101
C	-3.117743	-4.915417	3.338772
C	-2.134399	-4.374709	2.456233
C	-1.236089	-3.411394	2.946329

C -1.300947 -2.934150 4.255713
C -2.274169 -3.483637 5.107521
C -2.028169 -4.831717 1.095679
C -0.773016 -4.500599 0.401738
C 0.056921 -3.515605 0.939424
C 1.347695 -3.035972 0.431919
C 2.135805 -2.202703 1.254523
C 3.396800 -1.783224 0.835836
C 3.902533 -2.168580 -0.405377
C 3.128395 -2.994178 -1.252199
C 1.867204 -3.413134 -0.830658
O -0.272886 -2.924958 2.131621
O -2.903010 -5.559043 0.571999
O 3.604778 -3.376171 -2.459975
O 5.117378 -1.806340 -0.885159
O -0.460269 -5.221915 -0.647927
O -2.391295 -3.115570 6.391330
O -3.958464 -5.850337 2.887846
H -3.912054 -4.885902 5.343258
H -0.580078 -2.197706 4.613824
H 1.770994 -1.892807 2.232105
H 4.001893 -1.145564 1.487054
H 1.291631 -4.044185 -1.496595
H 4.542521 -3.116245 -2.565969
H 5.592674 -1.265767 -0.241341
H -1.861707 -2.299847 6.588064
H -3.768260 -5.940347 1.906820
H -0.470819 -5.054404 -2.200043
H -3.358210 -11.253311 2.565209
H -3.740505 -10.498895 -4.556601
H 3.347128 -6.133248 -5.478805
H 2.509197 -12.151017 1.931229

Ni-singlet

C -3.749272 -10.205833 2.496748
H -4.194355 -10.008898 3.483588
H -4.566033 -10.173334 1.762235
N -1.884460 -8.562129 3.067487
H -1.887249 -8.710372 4.086642
C -2.715668 -9.183700 2.155023
C -1.031519 -7.762810 2.383784
H -0.273351 -7.148334 2.860029
N -1.270970 -7.835327 1.081895
C -2.318985 -8.713228 0.925994
H -2.714783 -8.968795 -0.051202
C -3.195944 -9.558246 -4.857232
H -3.863914 -9.017402 -5.546024
H -2.285028 -9.853448 -5.400855
N -3.611462 -7.812592 -3.046601
H -4.578962 -7.606679 -3.339994
C -2.779516 -8.695667 -3.709465
C -2.918438 -7.221108 -2.045912
H -3.303487 -6.476843 -1.349382
N -1.675036 -7.689613 -2.024579
C -1.564797 -8.585630 -3.070635
H -0.625660 -9.064932 -3.325991
C 2.895647 -6.249012 -4.498121
H 3.242780 -5.451757 -3.820484
H 3.185874 -7.219712 -4.075287
C 1.369383 -6.159028 -4.637474
H 0.960774 -7.072541 -5.105670
H 1.052159 -5.306704 -5.252190
C 0.707932 -6.047794 -3.293806
O 1.047820 -6.768464 -2.353887
O -0.233532 -5.137249 -3.216668
C 2.972759 -11.626233 1.074545
H 3.363836 -12.428804 0.427606
H 3.841677 -11.049362 1.422186
N 1.081095 -11.240652 -0.566211
H 0.921242 -12.228901 -0.807515
C 2.024567 -10.752211 0.315987
C 0.335216 -10.215618 -1.021516
H -0.497219 -10.346272 -1.705725
N 0.762893 -9.074039 -0.506088
C 1.818074 -9.390372 0.327071
H 2.357688 -8.630566 0.883383
Ni -0.192031 -7.217461 -0.640250
O 1.456994 -6.469810 0.742775
O 1.213169 -5.764055 1.718758
C -3.193069 -4.508356 4.702298
C -3.161895 -4.947594 3.383240
C -2.189166 -4.413306 2.485451
C -1.277785 -3.457541 2.963758
C -1.319256 -2.979655 4.272995
C -2.284405 -3.521214 5.140150
C -2.123784 -4.849255 1.115487
C -0.894037 -4.499384 0.376978

C -0.027609 -3.542632 0.926095
C 1.254652 -3.059257 0.404231
C 2.055737 -2.241461 1.231828
C 3.317789 -1.828319 0.812646
C 3.814369 -2.208751 -0.433591
C 3.028183 -3.018443 -1.285598
C 1.763423 -3.427172 -0.865764
O -0.323241 -2.975412 2.135235
O -3.014737 -5.566720 0.608257
O 3.503175 -3.389907 -2.497672
O 5.029484 -1.856049 -0.916684
O -0.642040 -5.172763 -0.708825
O -2.380054 -3.149151 6.424343
O -4.017751 -5.872141 2.942871
H -3.927384 -4.914136 5.398837
H -0.589042 -2.246815 4.618734
H 1.699632 -1.939496 2.214831
H 3.930865 -1.201581 1.466723
H 1.177359 -4.045373 -1.533794
H 4.442417 -3.133618 -2.592990
H 5.528891 -1.352962 -0.260780
H -1.853293 -2.328483 6.608769
H -3.848571 -5.957117 1.957432
H -0.557758 -5.068568 -2.261297
H -3.344247 -11.216051 2.555752
H -3.709832 -10.464287 -4.536179
H 3.358107 -6.139947 -5.479091
H 2.502287 -12.149302 1.907085

Ni-triplet

C -3.767079 -10.223447 2.494955
H -4.214151 -10.032896 3.482222
H -4.583968 -10.198988 1.760202
N -1.903112 -8.580020 3.063054
H -1.893012 -8.738398 4.080501
C -2.745467 -9.190006 2.152770
C -1.059728 -7.770113 2.380375
H -0.293242 -7.160843 2.849405
N -1.316519 -7.824062 1.080803
C -2.365693 -8.700311 0.925350
H -2.771611 -8.944978 -0.050806
C -3.190089 -9.550939 -4.853102
H -3.854377 -9.014430 -5.548908
H -2.275530 -9.847607 -5.389808
N -3.619652 -7.798738 -3.051607
H -4.587096 -7.599185 -3.348614
C -2.782184 -8.682180 -3.706791
C -2.930217 -7.197608 -2.053262
H -3.323474 -6.450042 -1.365649
N -1.684867 -7.659444 -2.025720
C -1.569315 -8.561326 -3.065825
H -0.627721 -9.038220 -3.316654
C 2.894842 -6.260371 -4.517387
H 3.235355 -5.462594 -3.837368
H 3.188478 -7.229366 -4.093258
C 1.368061 -6.181906 -4.662494
H 0.967890 -7.106690 -5.115636
H 1.045785 -5.344189 -5.294570
C 0.705539 -6.051040 -3.321464
O 1.043774 -6.748308 -2.367485
O -0.241083 -5.138776 -3.266134
C 2.976057 -11.583897 1.084368
H 3.384254 -12.375978 0.435300
H 3.833928 -10.996551 1.442186
N 1.093910 -11.219021 -0.570363
H 0.949119 -12.208602 -0.814127
C 2.021777 -10.719689 0.323064
C 0.336424 -10.203217 -1.027836
H -0.488241 -10.341946 -1.720231
N 0.742972 -9.058729 -0.503085
C 1.794514 -9.361219 0.338920
H 2.313451 -8.590166 0.900296
Ni -0.191550 -7.197728 -0.618979
O 1.364706 -6.464371 0.693640
O 1.060382 -5.750186 1.676812
C -3.164301 -4.508621 4.715987
C -3.138110 -4.933646 3.391891
C -2.155112 -4.405789 2.501598
C -1.225056 -3.474107 2.991993
C -1.260471 -3.010852 4.304772
C -2.240682 -3.542212 5.164527
C -2.105430 -4.817681 1.126036
C -0.874348 -4.464452 0.377316
C 0.020771 -3.529473 0.948133
C 1.290571 -3.046256 0.413182
C 2.085456 -2.197956 1.218809
C 3.339506 -1.782435 0.783914

C 3.835778 -2.191101 -0.455019
C 3.059051 -3.036600 -1.282621
C 1.801770 -3.448309 -0.847134
O -0.259383 -2.994142 2.169397
O -3.006504 -5.507904 0.605803
O 3.536212 -3.438018 -2.482369
O 5.043821 -1.837249 -0.948178
O -0.651973 -5.099961 -0.719354
O -2.335326 -3.177150 6.449923
O -4.011488 -5.835009 2.941098
H -3.908937 -4.905822 5.406328
H -0.518633 -2.294931 4.660517
H 1.729751 -1.877537 2.196162
H 3.949802 -1.132740 1.417735
H 1.225054 -4.104334 -1.486692
H 4.473107 -3.176768 -2.591394
H 5.534951 -1.301209 -0.312148
H -1.814086 -2.353251 6.636560
H -3.846073 -5.915311 1.955882
H -0.570208 -5.056855 -2.324522
H -3.351314 -11.229298 2.553853
H -3.704265 -10.457488 -4.533947
H 3.361288 -6.148286 -5.496127
H 2.506380 -12.119606 1.909284

Ni-quintet

C -3.747963 -10.210768 2.491571
H -4.191381 -10.012567 3.478790
H -4.565532 -10.178236 1.757852
N -1.885623 -8.562540 3.057929
H -1.890595 -8.704650 4.077964
C -2.713325 -9.190658 2.147361
C -1.030273 -7.768018 2.371591
H -0.277206 -7.149414 2.850117
N -1.264246 -7.849761 1.068997
C -2.311511 -8.729486 0.916672
H -2.704654 -8.994255 -0.059382
C -3.220366 -9.573392 -4.869530
H -3.890228 -9.036349 -5.559265
H -2.308383 -9.865808 -5.412876
N -3.644134 -7.829694 -3.059673
H -4.610937 -7.624590 -3.356616
C -2.807984 -8.708606 -3.722774
C -2.955608 -7.239083 -2.055693
H -3.345003 -6.497987 -1.358708
N -1.711044 -7.703079 -2.034570
C -1.594554 -8.595019 -3.082615
H -0.653140 -9.071096 -3.335628
C 2.884837 -6.251228 -4.480760
H 3.228500 -5.450316 -3.805359
H 3.181250 -7.219718 -4.056936
C 1.356668 -6.172976 -4.618304
H 0.955332 -7.091534 -5.082164
H 1.033811 -5.323600 -5.234202
C 0.701849 -6.060955 -3.271751
O 1.009522 -6.819062 -2.348772
O -0.192685 -5.108728 -3.166719
C 2.967876 -11.614616 1.071107
H 3.367770 -12.414811 0.426671
H 3.831233 -11.030198 1.420253
N 1.085528 -11.248946 -0.583962
H 0.936577 -12.238975 -0.824953
C 2.015018 -10.750519 0.307097
C 0.332744 -10.232194 -1.046187
H -0.491881 -10.374091 -1.738277
N 0.741349 -9.085491 -0.526891
C 1.791660 -9.391351 0.317563
H 2.316681 -8.627915 0.882389
Ni -0.231621 -7.247014 -0.671880
O 1.497759 -6.452954 0.903340
O 1.282664 -5.715958 1.855175
C -3.184302 -4.485665 4.685805
C -3.136946 -4.926294 3.368009
C -2.153653 -4.392157 2.481268
C -1.250506 -3.431751 2.967788
C -1.309066 -2.952785 4.276545
C -2.281166 -3.497441 5.133234
C -2.062287 -4.842685 1.117399
C -0.816804 -4.503813 0.406679
C 0.025307 -3.525876 0.948710
C 1.312977 -3.046735 0.434035
C 2.103224 -2.209171 1.251208
C 3.364722 -1.794217 0.830127
C 3.870340 -2.189551 -0.407916
C 3.095539 -3.021163 -1.248424
C 1.833655 -3.434826 -0.825018
O -0.289028 -2.947404 2.149472

O -2.945506 -5.562673 0.598103
O 3.574966 -3.412061 -2.452418
O 5.084880 -1.832529 -0.891204
O -0.521699 -5.198793 -0.658283
O -2.390805 -3.127624 6.417074
O -3.985405 -5.854166 2.918851
H -3.924133 -4.892973 5.375574
H -0.584304 -2.218115 4.629859
H 1.740102 -1.893953 2.227585
H 3.970994 -1.152888 1.476575
H 1.259665 -4.075366 -1.481886
H 4.510765 -3.145742 -2.558144
H 5.568366 -1.300278 -0.246632
H -1.860608 -2.310952 6.609157
H -3.801197 -5.942653 1.936352
H -0.494636 -5.045578 -2.200401
H -3.343286 -11.221093 2.551113
H -3.729722 -10.480653 -4.544723
H 3.345906 -6.141832 -5.462347
H 2.498822 -12.140483 1.902683

Cu-doublet

C -3.778884 -10.264491 2.486068
H -4.223623 -10.076284 3.474674
H -4.599985 -10.252968 1.755913
N -1.944978 -8.586395 3.046260
H -1.929055 -8.745139 4.062784
C -2.775530 -9.215759 2.137581
C -1.127950 -7.750651 2.361364
H -0.373815 -7.118132 2.819864
N -1.388889 -7.807693 1.063451
C -2.412467 -8.713791 0.909274
H -2.811294 -8.970887 -0.067206
C -3.155085 -9.545177 -4.834857
H -3.812128 -9.009275 -5.538273
H -2.234941 -9.842586 -5.360926
N -3.612491 -7.785251 -3.051153
H -4.579928 -7.597826 -3.356768
C -2.761896 -8.670568 -3.687141
C -2.936998 -7.166899 -2.054212
H -3.342645 -6.408230 -1.387347
N -1.688014 -7.616449 -2.009715
C -1.556067 -8.533126 -3.035107
H -0.608750 -9.009557 -3.265366
C 2.862156 -6.221885 -4.497801
H 3.192793 -5.410097 -3.828911
H 3.160796 -7.180127 -4.052953
C 1.337835 -6.153882 -4.663535
H 0.943915 -7.096236 -5.086186
H 1.020128 -5.345710 -5.336304
C 0.638118 -5.968694 -3.345996
O 1.003643 -6.530334 -2.321908
O -0.397775 -5.150312 -3.405710
C 3.001835 -11.644134 1.118262
H 3.412006 -12.445220 0.480872
H 3.860991 -11.062938 1.482465
N 1.147884 -11.284835 -0.560994
H 0.987293 -12.275951 -0.784147
C 2.069053 -10.777228 0.334069
C 0.417972 -10.263567 -1.056181
H -0.399181 -10.410035 -1.757017
N 0.831700 -9.113153 -0.557377
C 1.860053 -9.413320 0.310082
H 2.380260 -8.634129 0.862049
Cu -0.238563 -7.205958 -0.603367
O 1.160948 -6.361333 0.526518
O 0.788328 -5.690873 1.544293
C -3.072489 -4.509832 4.705127
C -3.044616 -4.892651 3.367007
C -2.075685 -4.320961 2.487088
C -1.146872 -3.401866 3.008256
C -1.187724 -2.978708 4.330305
C -2.165947 -3.541832 5.177002
C -2.064089 -4.648617 1.091733
C -0.892730 -4.157880 0.290773
C 0.076275 -3.314150 0.949177
C 1.340699 -2.859292 0.416527
C 2.189683 -2.090017 1.253746
C 3.454642 -1.720962 0.820339
C 3.912089 -2.099286 -0.446425
C 3.081818 -2.862613 -1.306728
C 1.810303 -3.224649 -0.875913
O -0.166559 -2.892548 2.212500
O -2.941404 -5.348046 0.556519
O 3.523148 -3.229211 -2.528569
O 5.128660 -1.790096 -0.934967
O -0.765343 -4.570841 -0.878101

O -2.266436 -3.201675 6.466760
O -3.913068 -5.785422 2.895708
H -3.809663 -4.938884 5.384023
H -0.455121 -2.264215 4.705964
H 1.861240 -1.802974 2.251306
H 4.107361 -1.133015 1.471860
H 1.185756 -3.817761 -1.534400
H 4.477846 -3.037877 -2.635145
H 5.654935 -1.303963 -0.286551
H -1.764156 -2.368106 6.665087
H -3.751453 -5.846886 1.910471
H -0.754324 -5.013553 -2.493955
H -3.352213 -11.265486 2.549517
H -3.674281 -10.451459 -4.523162
H 3.335554 -6.120707 -5.474389
H 2.516860 -12.172385 1.939124

Cu-quartet

C -3.682586 -10.120876 2.468360
H -4.125665 -9.909171 3.453039
H -4.495374 -10.066327 1.730310
N -1.773319 -8.527293 3.043190
H -1.786116 -8.673574 4.062688
C -2.617617 -9.130103 2.128916
C -0.895729 -7.753336 2.363173
H -0.125140 -7.156207 2.840655
N -1.135715 -7.824910 1.060859
C -2.205971 -8.675531 0.900035
H -2.606190 -8.910840 -0.080184
C -3.281724 -9.637902 -4.928475
H -3.954740 -9.113739 -5.624747
H -2.371616 -9.937555 -5.471419
N -3.694123 -7.859188 -3.151199
H -4.658463 -7.647673 -3.451845
C -2.865587 -8.754387 -3.798172
C -2.994407 -7.258990 -2.156386
H -3.384751 -6.504870 -1.474205
N -1.754310 -7.726448 -2.125311
C -1.652761 -8.639214 -3.154969
H -0.718878 -9.134391 -3.401909
C 2.878590 -6.290479 -4.447371
H 3.217302 -5.487028 -3.772517
H 3.188093 -7.255630 -4.025877
C 1.349014 -6.231665 -4.577088
H 0.955914 -7.159237 -5.029721
H 1.010125 -5.391671 -5.197414
C 0.697565 -6.113366 -3.229912
O 1.027377 -6.861969 -2.300766
O -0.207321 -5.178159 -3.128620
C 2.988370 -11.634797 1.078307
H 3.370317 -12.442734 0.433101
H 3.863287 -11.068157 1.427743
N 1.119493 -11.220509 -0.584884
H 0.955248 -12.205698 -0.836358
C 2.052760 -10.749534 0.318225
C 0.384527 -10.185896 -1.036183
H -0.438879 -10.295188 -1.735584
N 0.811112 -9.056117 -0.493950
C 1.851026 -9.387806 0.350948
H 2.387794 -8.641598 0.927180
Cu -0.146137 -7.255743 -0.638139
O 1.830543 -6.339938 0.879188
O 1.524431 -5.798552 1.918171
C -3.201343 -4.494845 4.682376
C -3.142878 -4.949166 3.369426
C -2.150606 -4.426138 2.487013
C -1.248887 -3.464738 2.972429
C -1.316669 -2.972891 4.276765
C -2.298469 -3.505454 5.129132
C -2.044734 -4.895103 1.128107
C -0.790404 -4.569802 0.433070
C 0.046593 -3.592081 0.967983
C 1.336695 -3.108212 0.456766
C 2.125402 -2.275475 1.279321
C 3.389147 -1.859474 0.864127
C 3.899855 -2.252204 -0.372260
C 3.126208 -3.078403 -1.218457
C 1.860529 -3.489900 -0.802350
O -0.278570 -2.993023 2.158143
O -2.922324 -5.622538 0.609986
O 3.609544 -3.465858 -2.422232
O 5.115953 -1.895303 -0.852140
O -0.495610 -5.271834 -0.639034
O -2.418699 -3.123225 6.409067
O -3.987878 -5.880542 2.919956
H -3.948937 -4.892666 5.369431
H -0.590871 -2.238579 4.629601

H 1.758664 -1.959928 2.254344
H 3.991895 -1.219193 1.514800
H 1.284853 -4.119798 -1.468719
H 4.544130 -3.192627 -2.527767
H 5.612452 -1.394228 -0.192879
H -1.882146 -2.310936 6.601084
H -3.793295 -5.976219 1.938788
H -0.511490 -5.116238 -2.158307
H -3.307973 -11.142625 2.529674
H -3.786048 -10.540541 -4.583558
H 3.334285 -6.172444 -5.430467
H 2.507916 -12.149296 1.910485

Zn-singlet

C -3.773735 -10.251204 2.524071
H -4.217667 -10.070374 3.514200
H -4.592031 -10.217893 1.791290
N -1.914033 -8.606858 3.107878
H -1.914951 -8.761343 4.126629
C -2.748403 -9.218517 2.191963
C -1.058344 -7.805871 2.430149
H -0.299189 -7.194106 2.908736
N -1.298898 -7.867261 1.128451
C -2.351548 -8.739223 0.966536
H -2.749824 -8.985360 -0.013114
C -3.245619 -9.609314 -4.876890
H -3.911008 -9.077799 -5.575075
H -2.331517 -9.907864 -5.413263
N -3.677296 -7.844800 -3.090433
H -4.642660 -7.641485 -3.394783
C -2.838193 -8.732927 -3.737274
C -2.993074 -7.238383 -2.094374
H -3.387620 -6.485962 -1.413517
N -1.746494 -7.696983 -2.061791
C -1.627735 -8.608686 -3.094255
H -0.684958 -9.087286 -3.338279
C 2.909935 -6.226374 -4.521203
H 3.250688 -5.419351 -3.851275
H 3.211430 -7.188970 -4.087411
C 1.380914 -6.160546 -4.655467
H 0.985373 -7.081451 -5.118922
H 1.048705 -5.311558 -5.266731
C 0.744115 -6.057172 -3.299570
O 1.044330 -6.825921 -2.387884
O -0.128893 -5.081581 -3.173226
C 2.995049 -11.631642 1.106745
H 3.391909 -12.433759 0.463071
H 3.861306 -11.057450 1.464949
N 1.133305 -11.242381 -0.563131
H 0.978940 -12.229706 -0.810696
C 2.058852 -10.754445 0.338774
C 0.394458 -10.217417 -1.027694
H -0.424334 -10.346225 -1.729344
N 0.807122 -9.076678 -0.496575
C 1.846573 -9.393347 0.355255
H 2.369952 -8.631815 0.924812
Zn -0.256407 -7.266403 -0.650456
O 1.505642 -6.462214 0.907526
O 1.187388 -5.759918 1.870589
C -3.179926 -4.443611 4.637680
C -3.105488 -4.890653 3.323660
C -2.098892 -4.366191 2.457739
C -1.194903 -3.414607 2.960777
C -1.278905 -2.931143 4.265607
C -2.278657 -3.461600 5.100808
C -1.985135 -4.817616 1.097993
C -0.720815 -4.492874 0.411015
C 0.127739 -3.524416 0.971883
C 1.416715 -3.049147 0.465752
C 2.194734 -2.189615 1.273120
C 3.453357 -1.770611 0.849328
C 3.967211 -2.180437 -0.381878
C 3.204463 -3.033418 -1.212323
C 1.947381 -3.454853 -0.784113
O -0.207221 -2.942803 2.162835
O -2.860550 -5.530678 0.557913
O 3.685767 -3.438297 -2.409546
O 5.179261 -1.817673 -0.865074
O -0.420544 -5.190310 -0.644209
O -2.417569 -3.082799 6.378409
O -3.949853 -5.815345 2.860588
H -3.939721 -4.840912 5.311311
H -0.555203 -2.203178 4.634677
H 1.823489 -1.860732 2.242193
H 4.052099 -1.113378 1.486839
H 1.385379 -4.117156 -1.429942
H 4.617853 -3.163065 -2.529644

H 5.648590 -1.258856 -0.232367
H -1.878804 -2.274527 6.582571
H -3.746061 -5.910080 1.883352
H -0.409641 -5.023470 -2.205597
H -3.355987 -11.256761 2.573138
H -3.756756 -10.513759 -4.547066
H 3.370940 -6.123194 -5.503493
H 2.511852 -12.155257 1.931618

Zn-triplet
C -3.769912 -10.245983 2.520977
H -4.219396 -10.064752 3.508369
H -4.583759 -10.210582 1.783240
N -1.912999 -8.600342 3.114588
H -1.918587 -8.752757 4.133802
C -2.740914 -9.214876 2.195388
C -1.054448 -7.800156 2.439691
H -0.302092 -7.188106 2.928105
N -1.285465 -7.865406 1.136699
C -2.337036 -8.738378 0.971425
H -2.732124 -8.985079 -0.009331
C -3.258967 -9.615972 -4.888986
H -3.924912 -9.088060 -5.589127
H -2.343358 -9.912985 -5.423781
N -3.692750 -7.848476 -3.105153
H -4.657738 -7.643281 -3.410723
C -2.853818 -8.737024 -3.751460
C -3.011690 -7.246441 -2.105216
H -3.403531 -6.493495 -1.422920
N -1.766598 -7.709253 -2.070913
C -1.645481 -8.617992 -3.104986
H -0.702759 -9.097213 -3.347714
C 2.906627 -6.223283 -4.518050
H 3.248703 -5.415994 -3.849087
H 3.207297 -7.186295 -4.084425
C 1.377321 -6.155382 -4.651591
H 0.981327 -7.073139 -5.120617
H 1.047018 -5.301881 -5.257354
C 0.741615 -6.061032 -3.294269
O 1.033980 -6.850540 -2.395827
O -0.117958 -5.079034 -3.148963
C 2.990711 -11.627962 1.100244
H 3.383395 -12.430344 0.454314
H 3.859371 -11.056365 1.456860
N 1.124098 -11.229795 -0.564034
H 0.966820 -12.216302 -0.813711
C 2.053714 -10.747832 0.336516
C 0.387085 -10.200688 -1.022392
H -0.433042 -10.324769 -1.723127
N 0.803812 -9.062280 -0.488226
C 1.844912 -9.386391 0.358774
H 2.375761 -8.632866 0.931155
Zn -0.289893 -7.270074 -0.676026
O 1.595244 -6.443619 1.045693
O 1.292862 -5.758687 2.002929
C -3.184891 -4.446405 4.628099
C -3.111463 -4.894232 3.314321
C -2.108831 -4.366801 2.445662
C -1.209863 -3.409244 2.946232
C -1.293849 -2.924205 4.252075
C -2.286915 -3.459645 5.089665
C -1.986814 -4.828263 1.087561
C -0.720329 -4.510327 0.412798
C 0.113668 -3.535397 0.961317
C 1.409945 -3.061461 0.460407
C 2.185379 -2.202813 1.268963
C 3.444955 -1.780430 0.847319
C 3.962636 -2.187698 -0.382209
C 3.202769 -3.041126 -1.213214
C 1.945148 -3.465395 -0.787223
O -0.226804 -2.936949 2.146700
O -2.859712 -5.551239 0.552016
O 3.685648 -3.444377 -2.411229
O 5.175819 -1.821608 -0.864331
O -0.400376 -5.228741 -0.637803
O -2.423211 -3.084343 6.369648
O -3.951417 -5.825235 2.853408
H -3.939453 -4.847495 5.305407
H -0.571721 -2.193187 4.618498
H 1.811522 -1.873326 2.236795
H 4.039642 -1.120803 1.486195
H 1.384898 -4.124662 -1.437265
H 4.616210 -3.164377 -2.530959
H 5.639701 -1.258802 -0.231412
H -1.883970 -2.276859 6.574172
H -3.747214 -5.918661 1.875390
H -0.391079 -5.029848 -2.171526

H	-3.353620	-11.252108	2.570774
H	-3.766433	-10.520368	-4.553409
H	3.367736	-6.120785	-5.500363
H	2.508954	-12.151039	1.926299
